# Demyelination Lesions Do Not Correlate with Clinical Manifestations by Bordetella pertussis Toxin Concentrations

**DOI:** 10.3390/life12070962

**Published:** 2022-06-27

**Authors:** Maiara Carolina Perussolo, Bassam Felipe Mogharbel, Claudia Sayuri Saçaki, Dilcele Silva Moreira Dziedzic, Seigo Nagashima, Leanderson Franco de Meira, Luiz Cesar Guarita-Souza, Lúcia de Noronha, Katherine Athayde Teixeira de Carvalho

**Affiliations:** 1 Advanced Therapy and Cellular Biotechnology in Regenerative Medicine Department, The Pelé Pequeno Príncipe Research Institute, Child and Adolescent Health Research & Pequeno Príncipe Faculties, Curitiba 80240-020, PR, Brazil; perussolo10@gmail.com (M.C.P.); bassamfm@gmail.com (B.F.M.); claudiasacaki@gmail.com (C.S.S.); dilceledz@gmail.com (D.S.M.D.); 2 Laboratory of Experimental Pathology, Graduate Program of Health Sciences, School of Medicine, Paraná Pontifical Catholic University (PUCPR), Curitiba 80215-901, PR, Brazil; seigocap@gmail.com (S.N.); lnno.noronha@gmail.com (L.d.N.); 3 Experimental Laboratory of Institute of Biological and Health Sciences, Paraná Pontifical Catholic University (PUCPR), Curitiba 80215-901, PR, Brazil; leandersonfm@gmail.com (L.F.d.M.); guaritasouzalc@hotmail.com (L.C.G.-S.)

**Keywords:** experimental allergic encephalomyelitis, multiple sclerosis, histopathological, *Bordetella pertussis* toxin, clinical scores, animal model

## Abstract

Multiple sclerosis (MS) is an autoimmune disease of the central nervous system, characterized as an inflammatory demyelinating disease. Given the need for improvements in MS treatment, many studies are mainly conducted through preclinical models such as experimental allergic encephalomyelitis (EAE). This study analyzes the relationships between histopathological and clinical score findings at EAE. Twenty-three female *Rattus norvegicus* Lewis rats from 6 to 8 weeks were induced to EAE. Nineteen rats underwent EAE induction distributed in six groups to establish the evolution of clinical signs, and four animals were in the control group. *Bordetella pertussis* toxin (PTX) doses were 200, 250, 300, 350 and 400 ng. The clinical scores of the animals were analyzed daily, from seven to 24 days after induction. The brains and spinal cords were collected for histopathological analyses. The results demonstrated that the dose of 250 ng of PTX induced a higher clinical score and reduction in weight. All induced groups demonstrated leukocyte infiltration, activation of microglia and astrocytes, and demyelinated plaques in the brains in histopathology. It was concluded that the dose of 250 ng and 350 ng of PTX were the best choices to trigger the brain and spinal cord demyelination lesions and did not correlate with clinical scores.

## 1. Introduction

Multiple sclerosis (MS) is a neurodegenerative disease of the central nervous system (CNS), characterized as an inflammatory demyelinating disease with a diversity of clinical signs and symptoms [1], been the most frequently cause of neurological incapacity in young adults [2], since the age of the first manifestation of symptoms is from around 20 to 40 years old. This condition impacts 2.5 million people around the world [3]. In Brazil, based on the Multiple Sclerosis Atlas of 2013, it varies from 5.01 to 20 patients for every 100,000 people [4]. The risk factors involved in the onset of MS are unclear; however, in recent years, new evidence has shown the relevance of environmental and genetic factors and the interaction between both as a possible trigger of MS [5]. Examples of environmental factors include viral infections, smoke, and D vitamin deficiency [6]. MS is classified into different phenotypes according to their signs and symptoms. The first event is called Isolated Clinical Syndrome, an acute episode characterized by its effects on optical nerve, spinal cord, and brainstem. The isolated clinical syndrome can evolve into a chronical form, called Secondary Progressive MS, or remain as a relapsing–remitting disease [3,7]. Around 10% of MS patients are diagnosed with Primary Progressive MS [8]. The treatments available for relapsing–remitting MS lead to significant improvements, although the same cannot be said for the Primary Progressive form, which still lacks more effective therapies. The first line of treatment acts by decreasing the inflammatory activity and neurological incapacity [9]. Therapeutic targets are the inflammatory activity, neuroprotection, and remyelination [10]. The great challenge in preclinical studies is to remain close to the clinical manifestations of the studied disease, which is mainly achieved through using animal models [11], as in the case of experimental allergic encephalomyelitis (EAE). However, some aspects seem not be elucidated in this model.

The pathological mechanisms are the central point of discussion in the development of new therapies based on cell-therapy approaches. Before starting the essays on new therapies, it is mandatory to try a preclinical model. In this context, after the literature review, the concentrations of Toxin Pertussis were identified as the key to develop this EAE in rats because they can be explored by different concentrations. EAE is an animal model used to study demyelinating diseases due to its similarities with the MS’s mechanisms [12]. Neural and axonal degradation, demyelination, motor dysfunction of the lower limbs, blood–brain barrier rupture, infiltration of T CD4+ and CD8+ cells, microglia activation and the production of pro-inflammatory cytokines are examples of the findings using EAE [1]. The lesions induced in EAE demonstrate variations in size and white-matter regions, with demyelination plaques preferentially occurring in the optic nerve, periventricular regions, cerebellar peduncles, and spinal cord. In rodents, the clinical manifestations resulting from EAE, such as paralysis and loss of tail tone, were noted [13]. Silke Walter and cols (2006) described the role of CD14 (LPS receptor) in EAE for the first time, in agreement with the findings of the increased immunoreactivity of CD14 in biopsy and postmortem brain tissues of MS patients when compared to age-matched controls. This emphasizes CD14’s role in the pathophysiological mechanisms in both conditions [14,15]. In a study by Silke Walter and cols (2006), mice were induced to EAE. They used 300 ng pertussis toxin from Bordetella pertussis that was intraperitoneally administered per animal on days 1 and 3 post-immunization (p.i.), and CD14 deficiency was associated with increased inflammatory infiltrates in the CNS [14].

The MS and its animal model EAE, with activated lymphocytes and phagocytes infiltrations leading the inflammatory cascade events and causing the SNC disarrangement of their cytoarchitecture with demyelination processes, vary in size and white-matter regions, with demyelination plaques preferentially occurring in the optic nerve, periventricular regions, cerebellar peduncles, and spinal cord. In rodents, the clinical manifestations of EAE, such as paralysis and loss of tail tone, were noted [13]. The Tool-Like Receptors (TLRs) responsible for pathogen recognition and the induction of innate immune responses were related to regulating the activation of antigen-presenting cells and key cytokines in EAE, and the increased TLRs expression in the absence of any evidence of microbial presence could explain why some species where resistant to the induction of EAE, particularly TRL9 [16,17]. On the other hand, it was recently identified that Cxcl10^+^ and Saa3^+^ monocytic subsets were derived from early myeloid cell progenitors by Single Cell analysis and that the perspectives of therapeutic targets were both potential pathogenic triggers of CNS inflammation in MS findings [18]. The pathological mechanisms were the central point of discussion in the development of new therapies before starting essays on new treatments that explain these interests to better understand the preclinical model when looking for therapeutic targets. Guinea pigs, rats, and mice are animal models under genetic control, and the strains can be genetically susceptible or resistant [19]. Each component could play a role in the induction of MS.

After the literature review, it was identified that the concentrations of Toxin Pertussis could be the key to the development of EAE in rats, such as the Primary Progressive form of MS, which needs to be explored. The essential point of Toxin Pertussis is its effect on the blood–brain barrier opening; therefore, it facilitates the migration of pathogenic T cells that enhance cytokine production and the induction of lymphocytosis to the CNS and triggers the inflammatory cascade [20]. There are limited studies on this. Therefore, the present study aims to analyze the pathological findings in Lewis rats induced to EAE by different Bordetella pertussis toxin concentrations using histochemistry approaches to standardize this model in rodents, to find new EAE therapies in the preclinical phase of future studies.

## 2. Materials and Methods

### 2.1. Animals

All procedures were approved by the Ethical Committee on the Use of Animals (CEUA) of the Pequeno Príncipe Complex and numbered 038-2018. The 23 animals were *Rattus norvegicus*, Lewis’s lineage, females, from 6 to 8 weeks of age, weighing around 170 g. They were kept in polypropylene boxes, receiving food and water ad libitum, under a 12 h light–dark cycle and average temperature between 21 and 23 °C. The animals were from Campinas University and kept at Pelé Pequeno Príncipe Research Institute.

### 2.2. Experimental Design

From the 23 rats, 19 underwent EAE induction to establish the evolution pattern of this preclinical model for future therapy assays. The evolution analyses of clinical signs were used to determine the best dosage of *Bordetella pertussis toxin* (PTX) that could develop EAE by induction. Four animals were maintained as a control group (without induction). The animals had their weight and clinical score analyzed daily, from 0, 7 days post-induction to 24 days post-induction.

### 2.3. EAE Induction

The animals were injected with 200 µL of the emulsion containing: 100 µg of the MPB peptide (Sigma-Aldrich^®^, St Louis, MO, USA) dissolved in 100 µL of 1% PBS (Sigma-Aldrich^®^, St Louis, MO, USA) and emulsified in 100 µL of Freund’s Adjuvant Supplement (Thermo Fisher Scientific Inc, Waltham, MA, USA), which contains *Mycobacterium butyricum* to increase the immune response, for immunization by subcutaneous injection on the dorsal region (100 µL) on both sides. At the point of induction and 48 h later, they received an intraperitoneal injection of *Bordetella pertussis* toxin (Sigma-Aldrich^®^, St Louis, MO, USA) at different concentrations: 200 ng (n = 4), 250 ng (n = 4), 300 ng (n = 4), 350 ng (n = 4) or 400 ng (n = 3) per dose. The control group submitted to the same injection procedures mentioned above, but using PBS (n = 4).

### 2.4. Clinical Signs of EAE

To assess the degree of EAE severity in a murine model, the animals were classified according to the clinical score scale described by Kerfoot and Kubes (2002) [21], based on motor clinical signs, according to Table 1.

### 2.5. Euthanasia

After the experiment, all animals were euthanized. First, each animal was anesthetized with a solution of ketamine (50 mg/kg) (Syntec, Tamboré, SP, Brazil) and xylazine (10 mg/kg) (Syntec, Tamboré, SP, Brazil), which was administered intraperitoneally. After anesthesia was confirmed, the animals were euthanized with a lethal dose of thiopental and then perfused to prepare the specimen for histopathological analysis. The perfusion was conducted with 4% formaldehyde solution and 0.1 mol/L of phosphate buffer (PBS). After complete perfusion, the brain, spinal cord and sciatic nerve were collected from each animal.

### 2.6. Histopathological Analysis

The collected tissues were stored in an 8% formaldehyde solution (Dinâmica Química Contemporânea LTDA, São Paulo, SP, Brazil) at room temperature. Then, the samples were dehydrated with alcohols at 70%, 80%, 90% and 100%, diaphanized in xylol and embedded in paraffin. The blocks were cut into 5-μm slices for histochemical techniques with Hematoxylin and Eosin (H&E) (Harris Hematoxylin: NewProv^®^, Pinhais, PR, Brazil; Eosin: BIOTEC Analytical Reagents^®^, Lages, SC, Brazil) and Luxol fast-blue (Sigma-Aldrich^®^, St Louis, MO, USA) techniques. Brains and spinal cords were evaluated to identify the aspects of demyelination and neuroinflammation score described by Racke et al., (1994) [22] (Table 2).

### 2.7. Statistical Analysis

For statistical analysis, One-Way ANOVA with Tukey’s post hoc was performed for parametric data and Kruskal–Wallis with Dunn’s post hoc for non-parametric data, with a significance level of 5% (*p* < 0.05), with the aid of the GraphPad Prism 7 program. Data were presented as mean ± standard error of the mean or median.

## 3. Results

### 3.1. Clinical Signs

**Figure 1:**
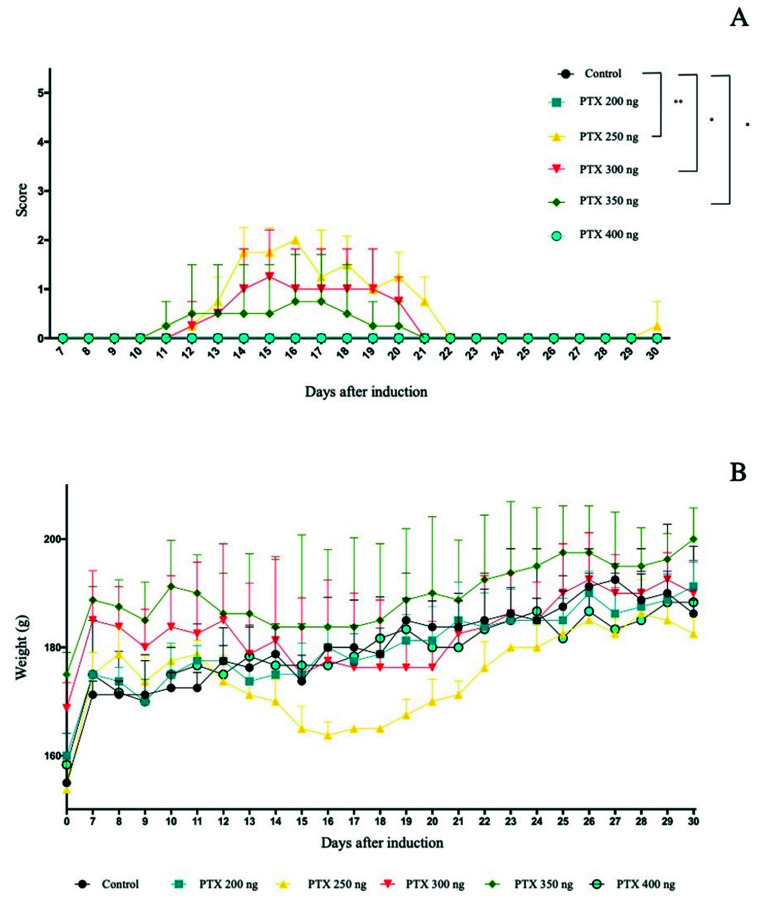
Evaluation of the score of clinical signs of EAE. Results are expressed as median. Statistical differences when *p* < 0.05 (*) and *p* < 0.001 (**). (**A**); weight variation (g) during the experiment days. Results expressed as mean ± standard error (**B**).

During the experiment, the animals’ clinical response to EAE induction was analyzed daily. The PTX 250 ng, PTX 300 ng and PTX 350 ng groups differed from the control. The score variation during the experiment can be seen in Figure 1A. The onset of clinical signs was observed on day 12 after induction and the peak occurred on day 15 after induction. 

### 3.2. Weight as a Clinical Sign

In addition to the score, animals were weighed daily. The weight (expressed as mean ± standard error) observed for the groups for 30 days was 180.15 ± 0.10, 179.95 ± 0.46, 174.7 ± 0.69, 182.75 ± 0.75, 189.6 ± 0.84 and 179.26 ± 0.84, respectively, for the control, PTX 200 ng, PTX 250 ng, PTX 300 ng, PTX 350 ng and PTX 400 ng groups (Figure 1B).

**Figure 2:**
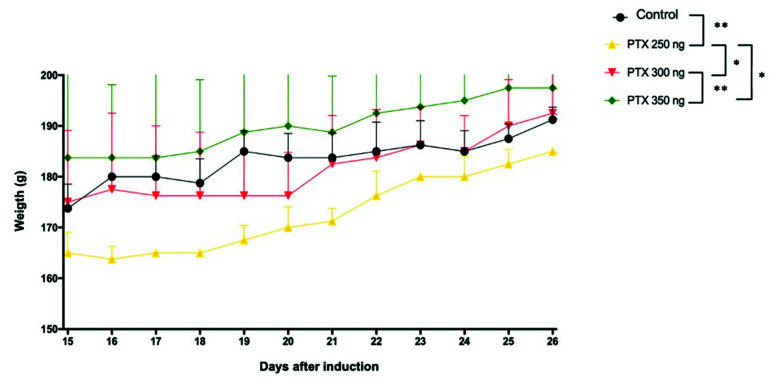
Analysis of weight (g) of the control, PTX 250, 300, and 350 groups during the peak period and recovery from EAE. Results expressed as mean ± standard error, *p* < 0.05 (*) and *p* < 0.001 (**).

Comparing the weight of the three groups that showed clinical signs (PTX 250, 300, and 350 ng) versus control, the peak in clinical signs occurred 15 h after induction until complete recovery and 26 h after induction. A statistically significant difference (*p* < 0.001) was observed between the vehicle control and PTX 250 groups (Figure 2). 

### 3.3. Histopathological Analysis 

After euthanasia, spinal cord and brain were collected for histopathological analysis. Then, slides were stained in H&E and Luxol Fast blue.

**Figure 3:**
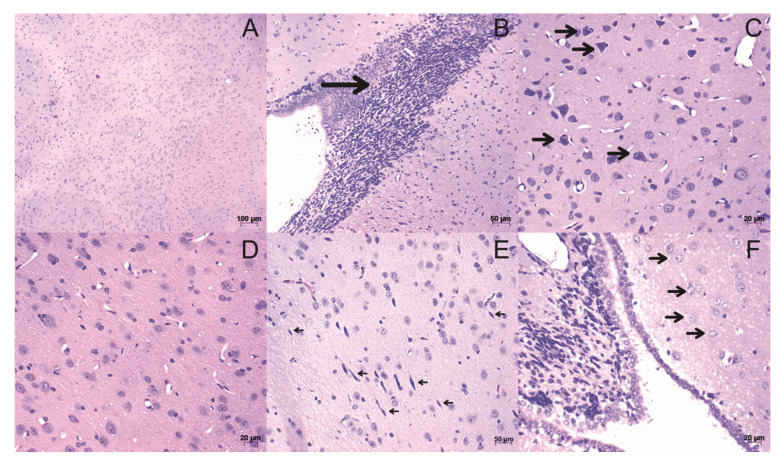
Histological sections of brain stained with H&E. (**A**,**D**) Tissue from control animals, with a homogeneous distribution of brain cell types. (**B**) Leukocyte infiltrations (arrow). (**C**) Neurons on cell death: Observing neurons accompanied by leukocytes (arrows). (**E**) Activation of microglia (arrows). (**F**) Astrocyte activation (**arrows**). The images were obtained from optical microscopy (Carl Zeiss Microscopy, Zeiss, Jena, Germany). Scale bar, 50 µm.

In the slides of brain tissue stained with H&E, the animal findings induced in the model included leukocyte infiltrates (Figure 3B), the presence of cell death in neurons (Figure 3C), activation of microglia (Figure 3E), and astrocytes (Figure 3F) in all tested PTX concentrations when compared to the control (Figure 3A,D).

**Figure 4:**
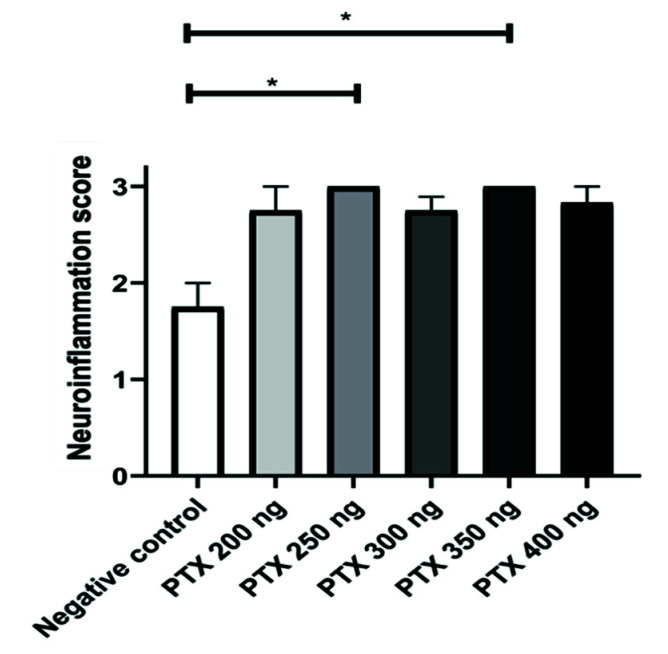
Neuroinflammation score of brain. Results are shown as mean ± standard error and statistically significant differences occur when *p* < 0.05 (*).

The neuroinflammation score was determined from the brain histopathological findings. The group with the lowest index of neuroinflammation was the negative control, as expected. In addition, the PTX 250 ng and PTX 350 ng groups showed statistically significant differences (*p* < 0.05) to the control, showing an increase in neuroinflammation (Figure 4).

**Figure 5:**
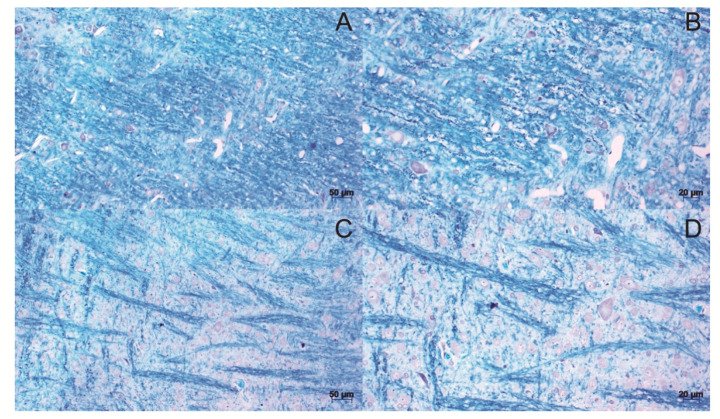
Histological sections of brain stained with Luxol fast blue. Tissue from control animals, with homogeneous distribution of myelin fibers (**A**,**B**). demyelinated plaques, present in the brain of animals induced to the model (**C**,**D**). The images were obtained from optical microscopy (Carl Zeiss Microscopy, Zeiss, Jena, Germany). Scale bar, 50 µm.

For the slides stained with Luxol Fast blue, it was possible to observe the loosening of the myelin fibers in all the induced groups, forming demyelinated plaques (Figure 5C,D) compared to the control (Figure 5A,B).

**Figure 6:**
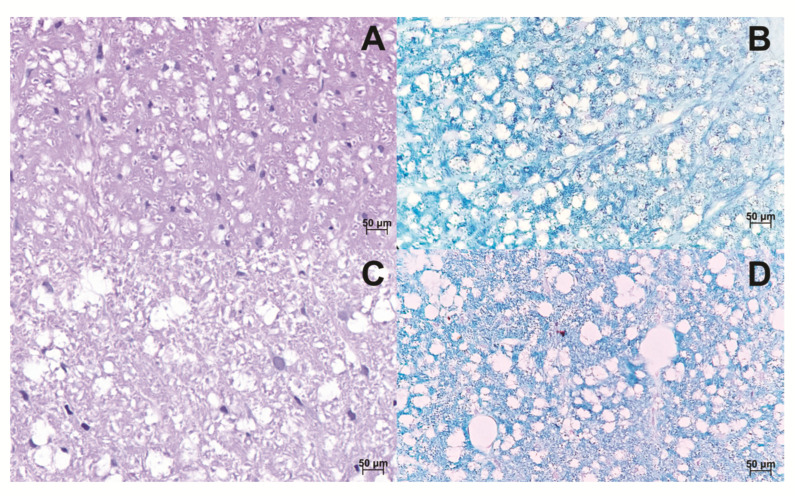
Histological sections of the Spinal cord. The spinal cord of control animals was stained in HE and Luxol fast blue, respectively (**A**,**B**). The spinal cord of animals induced to the EAE model, stained in HE and Luxol fast blue, respectively. It is possible to observe the loosening of the myelinated fibers by increasing the axonal space (**C**,**D**). The images were obtained from optical microscopy (Carl Zeiss Microscopy, Zeiss, Jena, Germany). Scale bar, 50 µm.

The spinal cord was also analyzed, focusing on the observation of myelin fibers. When compared to the control, induced animals demonstrated a loosening of the myelin fibers in the tissue in all tested PTX concentrations (Figure 6).

## 4. Discussion

The results of this study point to the efficiency of the experimental allergic encephalomyelitis model under the established conditions. The EAE model has been used in several studies involving the analysis of demyelination. In this study, the results obtained from the induction of the Lewis rats model follow those in the literature. Histopathological aspects such as leukocyte infiltration, microglia activation, and the presence of demyelinated plaques are classic demonstrations of induction [23,24]. Thus, the indirectly proportional relationship between clinical score increases, as well as the reduction in the weight of induced animals [24,25]. However, in EAE, the pathological findings are not uniform and depend on the animal species and the trigger protein. For example, in Lewis rats induced with EAE, the demyelination plaques are poorly defined, and inflammation is not extensive in brain tissue; such differences could explain the dissociation in clinical scores [26].

The experimental allergic encephalomyelitis induced by myelin basic protein exhibits an acute episode of clinical signs, followed by a partial or total recovery [27]. During the EAE induction protocol, doses of Bordetella pertussis toxin (PTX) were used after applying the peptide of choice to attenuate the presented clinical signs [28]. PTX has demonstrated the ability to increase the permeability of the blood–brain barrier, which induces the production of pro-inflammatory cytokines such as IL-12, acts on the activation of dendritic cells, and promotes the activation of IL-B1. The activation of IL-B1 also assists in the activation and recruitment of leukocytes Th1 and Th17 in brain tissue and increases the regulation of adhesion molecules in brain blood vessels [29,30]. In the present study, only animals induced in groups receiving a 250, 300, and 350 ng/dose of PTX showed clinical score manifestations with a consequent weight reduction, suggesting that the relationship between clinical signs and PTX concentration is not dose-dependent for the different tested concentrations. Despite these data, an interesting finding was the histopathological characteristics of this model in all induced animals, even those that did not show clinical motor signs. In published studies, it is possible to identify the different doses of PTX used in the EAE model and, consequently, different results were found. Toader et al. (2018) used a 150 ng/dose of PTX and observed the animals using the clinical score and weight parameters. After 34 days of EAE induction, the brain and spinal cord were collected and analyzed by histopathological techniques [31]. The classic demyelination plaques and leucocyte infiltration were not found, but alterations in myelin fibers, an increase in clinical score and a decrease in weight were observed. On the other hand, Zhang et al. (2018) used 500 ng/dose of PTX and described only leukocyte infiltration, resulting in a histopathological analysis [32]. These data demonstrate the similar aspects observed in the multiple sclerosis clinics, where demyelinated lesions were observed and detected in magnetic resonance images in asymptomatic patients [33,34]. These different histopathological findings suggested that each animal species has a different immunological response within the murine, in accordance with Gibson-Corley et al. (2016) [33]. The limitations of this study are that immunoreactivity was not analyzed; however, it emphasized morphology in the pathological findings based on histochemistry. In the future, these immunoassays will be performed to evaluate new EAE therapies [14,15]. This study also demonstrated the need to develop the pre-clinical model as a pilot before starting therapeutic trials, being the strong motivation of this study.

## 5. Conclusions

For this preclinical model of *Rattus norvegicus*, Lewis’s lineage, the 250 ng and 350 ng Bordetella pertussis toxin concentrations are the best choices to trigger demyelination lesions in the brain and spinal cord, as demonstrated by histopathological analysis. This study emphasizes that the EAE does not correlate with clinical scores and could be an excellent preclinical model for screening therapies in MS. The similarities of their findings explain: dissociation of the clinical score and the presence of demyelinating lesions by magnetic resonance imaging, as described in MS.

## Figures and Tables

**Table 1:** Clinical score scale to assess the degree of severity in Experimental Autoimmune Encephalopathy.

Score	Motor Clinical Signs
0	No signs
1	Tail paralysis
2	Tail paralysis and weakness in hind legs
3	Hind leg paralysis
4	Hind leg paralysis and weakness in the anterior legs
5	Total paralysis of the animal

**Table 2:** Neuroinflammation score proposed by Racke et al., (1994) [22].

Score	Histopathological Findings
0	No inflammatory cells
1	A few scattered inflammatory cells
2	Organization of inflammatory infiltrates around blood vessels
3	Extensive perivascular cuffing with extension into adjacent parenchyma, or parenchymal infiltration without obvious cuffing

## Data Availability

Not applicable.
